# The Potential Roles of Cervical Plexus Abnormalities in Occipital Neuralgia: An Anatomic Variant Explored

**DOI:** 10.3390/diagnostics12010139

**Published:** 2022-01-07

**Authors:** Mitchell H. Mirande, Heather F. Smith

**Affiliations:** 1 Arizona College of Osteopathic Medicine, Midwestern University, Glendale, AZ 85308, USA; mmirande76@midwestern.edu; 2 Department of Anatomy, College of Graduate Studies, Midwestern University, Glendale, AZ 85308, USA; 3 School of Human Evolution and Social Change, Arizona State University, Tempe, AZ 85287, USA

**Keywords:** accessory nerve, lesser occipital nerve, occipital neuralgia, accessory nerve impingement, headache disorder

## Abstract

Occipital neuralgia (ON) is a condition defined as a headache characterized by paroxysmal burning and stabbing pain located in the distribution of the greater occipital nerve (GON), lesser occipital nerve (LON), or third occipital nerves (TON). This condition can be severely impairing in symptomatic patients and is known to have numerous etiologies deriving from various origins such as trauma, anatomical abnormalities, tumors, infections, and degenerative changes. This study reports four cases of a previously undescribed anatomical variant in which the (spinal) accessory nerve (SAN) fuses with the LON before piercing the sternocleidomastoid (SCM). The fusion of these two nerves and their route through the SCM points to a potential location for nerve compression within the SCM and, in turn, another potential source of ON. This anatomical presentation has clinical significance as it provides clinicians with another possible cause of ON to consider when diagnosing patients who present with complaints of a headache. Additionally, this study explores the prevalence of piercing anatomy of the LON and GAN and discusses their clinical implications.

## 1. Introduction

Occipital neuralgia (ON) is defined as a paroxysmal burning or stabbing pain located in the posterior scalp along the distributions of the greater occipital nerve (GON), lesser occipital nerve (LON), or third occipital nerves (TON) [1,2,3,4,5]. It occurs unilaterally in around 85% of patients [1,6], and a Dutch study conducted in 2009 reported an incidence of 3.2% [7]. ON pain, which is often accompanied by tenderness over the nerve distribution, can be severely debilitating for patients and requires a clinical intervention that varies depending on the severity and etiology [3,4,5]. Due to the varied etiology associated with this condition, it is important for clinicians to identify the correct source of pathology in order to choose the most effective treatment modality. ON has been treated using a variety of approaches, from less invasive techniques such as nerve blocks and medication to more invasive methods such as denervation [2].

In typical anatomical examples, the lesser occipital nerve (LON) is derived from the anterior rami of the C2 and C3 spinal nerves. It travels superiorly along the posterior margin of the sternocleidomastoid muscle to the occipital region [8]. The LON penetrates the deep cervical fascia near the cranium and continues superiorly where it communicates with the GON and provides cutaneous sensory innervation to the skin [8]. The LON has three branches: the mastoid, auricular, and occipital branches, which innervate the lateral region of the head posterior to the ear and the superior surface of the ear [8].

The greater occipital nerve (GON) originates from the medial division of the posterior ramus of the C2 spinal nerve [8]. It recurs between the C1 and C2 vertebrae, where it then courses between the inferior capitis oblique and semispinalis capitis muscles from below the suboccipital triangle [8]. In most cases, the GON is found to pierce the semispinalis capitis muscle but, in other cases, may pierce the inferior oblique or trapezius [8]. The GON then travels through the aponeurotic fibrous layer of the trapezius and sternocleidomastoid muscles to reach the scalp and superior nuchal line [8]. Additionally, the GON travels alongside the occipital artery after passing through the semispinalis capitis muscle [8]. The GON innervates the skin on the back of the scalp up to the vertex of the skull, the ear, and the skin just superior to the parotid gland [8].

The (spinal) accessory nerve (SAN) origin has a spinal portion as well as a cranial root portion [9]. The spinal portion originates from the roots of C6–C7 then travels superiorly through the foramen magnum, where it then courses along the floor of the posterior cranial fossa, where it joins with the small cranial nerve roots emanating from the medulla oblongata [9]. Following its origin, the SAN exits the skull through the jugular foramen along with cranial nerves X and IX [9].

While occipital neuralgia has been frequently reported in the literature, there is often no clear etiology present [5] or agreement on treatment modality [3]. This study analyzes four cadaveric specimens with a unique anatomical presentation relating to the (spinal) accessory nerve and the lesser occipital nerve fusing and piercing the SCM as they travel to their location of innervation. Understanding this morphology will help provide another potential etiology to consider for ON, which will help clinicians approach this pathology with more information.

## 2. Materials and Methods

### 2.1. Samples

A total of 70 cadaveric specimens from the anatomy teaching laboratory at Midwestern University were included in this study. Thirty-eight males and thirty-two females were analyzed during the period between 2016 to 2019. The analysis was performed following student dissection of the cadavers, which consisted of cleaning the anterior and posterior triangles of the neck and reflection of the sternocleidomastoid muscle. An assessment of the sensory cervical plexus nerves, “spinal” accessory nerve (SAN), and sternocleidomastoid (SCM) was conducted on both sides of the cadaver prior to data collection. Further dissection was performed to open the visual field, allowing for nerve fibers to be followed from their point of origin to their target territory. Cadavers with damaged cervical plexus nerves, SANs, and SCMs were excluded from consideration. However, specimens were documented if at least one of the relevant nerves was intact. This study was determined to be IRB-exempt by the Midwestern University Institutional Review Board (#AZ 1354).

### 2.2. Data Collection and Analysis

**Figure 1:**
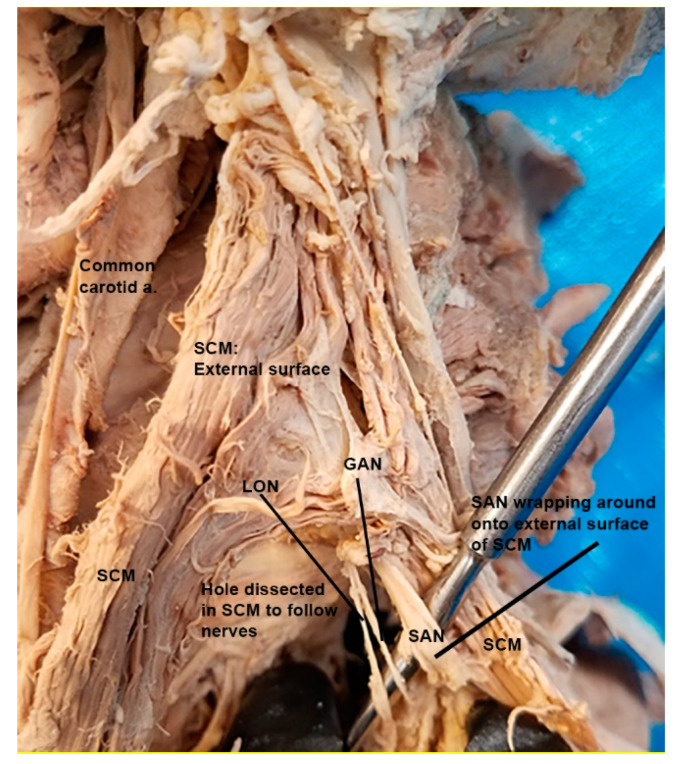
Dissection photo showing the spinal accessory nerve (SAN) as it pierces the sternocleidomastoid (SCM) and then wraps back superiorly towards the base of the scalp. A separation of the SCM was performed to allow for tracking and visualization of the course of the SAN as it traveled through the muscle.

On each cadaver, the sensory branches of the cervical plexus nerves and the SAN were traced from their origin in the deep neck to their target territory. Since the anatomy of the nerve branches was often discovered to vary bilaterally, each side of the cadaver was evaluated and scored separately. The presence of LON or GAN piercing of the SCM was noted. Additionally, the SAN was traced along its route deep to the SCM and the presence of any recurrent piercing was noted, in which fibers from the SAN traveled through the muscle belly and emerged superficially to course superiorly (Figure 1). It was also noted whether or not the SAN fused with any superiorly oriented sensory branches of the cervical plexus (LON, GAN) prior to piercing the SCM. Using SPSS 27 (IBM Corp.), a chi-squared test was performed to evaluate whether significant differences existed in variant frequencies between the sexes. 

## 3. Results

### 3.1. Spinal Accessory Nerve and Lesser Occipital Nerve Piercing Fusion

**Figure 2:**
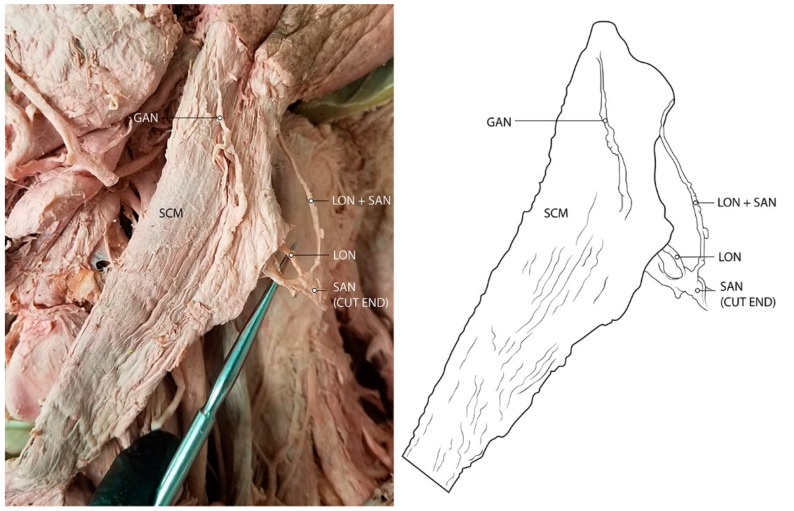
Cadaveric photo of connection between spinal accessory nerve (SAN) and lesser occipital nerve (LON), both of which pierce through the sternocleidomastoid. In this individual, the GAN also pierces the SCM, but does so separately from the SAN. Abbreviations: GAN = greater auricular nerve; LON = lesser occipital nerve; SAN = spinal accessory nerve; SCM = sternocleidomastoid.

**Figure 3:**
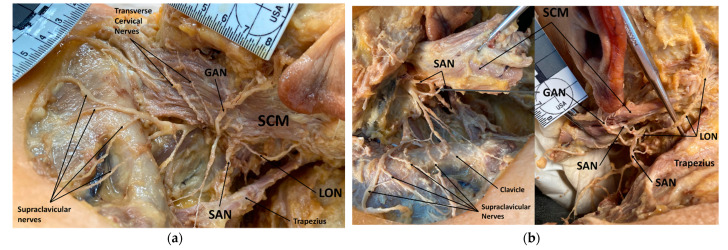
Cadaveric photo of left cervical and auricular regions showing variant with spinal accessory nerve (SAN) sending a small branch to join the lesser occipital nerve (LON): Superficial view (**a**) and deep view (**b**). Abbreviations: GAN = great auricular nerve; LON = lesser occipital nerve; SAN = spinal accessory nerve (CN XI); SCM = sternocleidomastoid.

Out of 106 cadaveric samples with intact SAN and LON, three specimens (on four sides) presented with the unique anatomical variant of the SAN fusing with the LON as they pierce the SCM and travel to the target territory of the LON in the occipital region of the scalp (Table 1; Figure 2). Two of the cadavers presented unilaterally (2F), and only one possessed the condition bilaterally (F). In an additional specimen, the SAN sent a small branch to meet the LON (Figure 3).

### 3.2. Lesser Occipital Nerve Piercing Sternocleidomastoid Alone

Out of 117 cadaveric sides with an intact LON, 68 had a LON that pierced the SCM (27F/41M) on its way superiorly. Both LONs on a single cadaver were recorded as separate data points. Out of those 117 LONs, 32 demonstrated a bilateral presentation of the piercing morphology (16 cadavers, 6F/10M).

### 3.3. Greater Auricular Nerve Piercing Sternocleidomastoid Alone

Out of 112 cadaveric sides with an intact GAN, 29 had a GAN that pierced the SCM (12F/17M) on its course towards the ear. Both GANs on a single cadaver were recorded as separate data points. Out of those 112 GANs, eight were a part of a bilateral piercing presentation (four cadavers, 2F/2M).

### 3.4. Lesser Occipital and Greater Auricular Nerve Piercing Sternocleidomastoid

Out of 102 cadaveric sides with an intact LON and GAN, 25 had both a LON and GAN that pierced the SCM (11F/24M) on the same side. Both LON and GAN pairs were measured as separate data points.

### 3.5. Statistical Analyses

No significant differences were revealed in frequency of variants between the sexes.

## 4. Discussion

### 4.1. Clinical Implications of Spinal Accessory Nerve Fusion with Lesser Occipital Nerve

In this study, there were four presentations of a unique nerve branching pattern with potential clinical implications for a variety of conditions. In four cadaveric specimens, the LON fused with the SAN as it pierced through the SCM and traveled towards its location of cutaneous innervation in the occipital region. This anatomic finding, to our knowledge, has never been reported in the literature and could result in a variety of different symptoms.

A recent study (Amirlak, 2020), discussed the relationship between the LON and the SAN in the neck concerning surgical procedures [10]. They reported that the LON and SAN have a close relationship and that consequently injuring the SAN during a LON procedure would be quite easy and could result in dramatic effects on a patient’s long-term shoulder movements [10]. As determined in our study, the close anatomical relationship between the LON and SAN can reach a point where they fuse and pierce the SCM. This situation could lead to compression and resulting symptoms in these patients. As shown in a variety of studies, the LON plays a role in multiple conditions relating to headache disorders or occipital pain such as occipital neuralgia, migraines, and cluster headaches [11]. Understanding this anatomical presentation could lead to a better diagnosis of the previously mentioned conditions, as well as a more effective treatment for patients. 

Typically, the SAN exits the jugular foramen at the base of the skull and travels towards the SCM where it dives into the muscle [12]. It then travels subcutaneously along the floor of the posterior cervical triangle where it reaches its final destination to innervate the trapezius [12]. The SAN, although traveling superficially through the neck, is injured less often than one might expect [12]. However, the presentation in which the LON fuses and travels with the SAN is the most likely cause of symptoms of LON compression. As seen in Figure 1, the LON may pierce the SCM (with the SAN) and then reverse course superiorly to reach its destination in the occipital region. This anatomy points to an additional point of LON compression which could contribute to symptoms of occipital pain, migraines, or other headache disorders. 

### 4.2. Clinical Implications of Lesser Occipital Nerve Piercing and Compression

The lesser occipital nerve (LON) has been an increasing point of interest in clinical literature for studies attempting to uncover the root cause of occipital pain. Mounting evidence supports its potentially significant role in primary headache disorders such as migraines, trigeminal neuralgia, and cluster headaches [11]. The LON is a part of the cervical plexus and derives its origin from the anterior ramus of the C2 nerve and sometimes receives contributions from the C3 nerve [2]. In normal anatomy, the LON travels rostrally and dorsally as it winds around the inferior border of the sternocleidomastoid (SCM) [2]. It continues towards the mastoid process, and at the level of the cranium, it pierces the deep cervical fascia beginning to work its way across the posterior edge of the SCM insertion and into the superficial fascia of the scalp [2]. Once in the superficial fascia of the scalp, the LON splits into the auricular, mastoid, and occipital branches [2]. The auricular branch travels anteriorly and superiorly to supply the upper medial region of the auricle and winds up communicating with the posterior branch of the great auricular nerve (GAN). In some cases, the auricular branch of the LON can originate from the greater occipital nerve (GON). The mastoid branch of the LON supplies the area over the mastoid process and tends to be smaller than the other branches. The occipital branches ramify superior to the mastoid process to supply the scalp along with the help of the GON [2]. 

Along its route from the C2 anterior ramus to the superficial fascia of the scalp, there are a few potential locations where the LON may become entrapped or compressed. A 2005 cadaveric study reported LON compression to occur in approximately 13.3% of their cases [13]. In our study, 68 out of 117 cadaveric samples (58%) were found to have a LON that pierced the SCM. This number is drastically higher than previous studies and points to another potentially critical location of compression of the LON. In a 2016 study, three zones of potential LON compression were investigated and the course of the LON was found to vary within zones [14]. Zone 1 was defined as the point in which the LON emerged behind the SCM, zone 2 was at the midpoint of the LON, and zone 3 was the position at which the LON crossed the nuchal line [14]. However, there was no mention of the LON piercing the SCM in any cases or the occurrence rate of compression in each zone. 

In recent studies, the upper cervical nerves C1–C3 have been investigated for their potential contribution to headache disorders [11]. A 2013 study showed that stimulation of the C1 nerve evoked pain in the periorbital and frontal areas of patients with migraines, but in the occipital and cervical regions in patients without migraines [11]. Additionally, stimulation of both the C2 and C3 nerves resulted in pain in the occipital and cervical regions [11]. Since the C2 and C3 spinal nerves give rise to the LON, GAN, and also the greater and third occipital nerves, this study points to the potential of these nerves to play a role in the presentation of migraines and occipital pain [6,11]. 

Our study shows that the LON piercing the SCM was a common finding within our cadaveric population and could be another potential location of compression, which is a clinically relevant contributor to the symptomology in many patients experiencing occipital pain [14]. Clinicians should be aware of this presentation as they attempt to diagnose and treat the variety of headache disorders. 

### 4.3. Clinical Implications of Greater Auricular Nerve Piercing and Compression

The GAN is a branch of the cervical plexus that originates from the anterior rami of the C2 and C3 spinal nerves [15]. It is a cutaneous branch that innervates the skin over the external ear, angle of the mandible, and parotid gland. It may share fibers with the auricular branch of the vagus nerve, the posterior auricular branch of the facial nerve (CN VII), the LON, or the auriculotemporal nerve of the trigeminal nerve [15]. The GAN has occasionally been implicated in causing facial pain that may be accompanied by other symptoms related to the cervical plexus [15]. 

Our study showed that the GAN pierced the SCM in 29 of 112 samples analyzed (25.9%). To our knowledge, the prevalence of this anatomic presentation has not been previously reported. The presence of the GAN piercing the SCM points to a potential location of nerve compression which could contribute to the presentation of symptoms in patients. As mentioned above, the study by Johnston showed that the C1–C3 upper cervical nerves, when stimulated, resulted in pain in the occipital and cervical regions [11]. Since the GAN originates from within these spinal nerves, its injury or compression could result in migraine and occipital pain symptoms [6,11].

### 4.4. Diagnosing Occipital Neuralgia

The process of diagnosing different types of headaches or facial pain is complicated by the overlapping of signs and symptoms that are typically considered at first presentation [7]. Being able to differentiate between the variety of origins of occipital pain is crucial to physicians when selecting the correct treatment modality. 

One of the diagnostic criteria for ON is the relief of occipital pain following the use of local anesthetic [16]. This helps explain the fact that the local anesthetic nerve block of the greater occipital nerve (GON) is the most commonly used tool for the diagnosis of ON [16,17]. Although it is the most commonly used, there is no standardized approach to this technique for physicians to follow [17]. The variability in technique can lead to varying results and to the potential of misdiagnosing the root cause of the occipital pain. Many studies have been conducted to try and locate the precise positions of the GON and LON using anatomical landmarks; however, due to variability in anatomy this approach may lead to collateral anesthesia, lessening the accuracy of the diagnosis [18]. Additionally, the conventional injection technique requires a relatively high volume of anesthetic which can lead to the unnecessary blockage of other nerves in the region (LON or TON), which would also lead to a less precise diagnosis of ON [17,18]. 

Recently there has been a growing interest in using ultrasound-guided approaches for these anesthetic injections due to the evidence showing improved patient outcomes [18]. The incidence of collateral anesthesia of nearby nerves is unknown but the use of ultrasound and small volumes of anesthetic is likely to reduce the risk [18]. Using ultrasound to guide anesthetic injections for both diagnosis and treatment of headache disorders such as ON can help physicians accurately identify the nerve that is causing the problem without affecting surrounding nerves or structures. In our study, we report instances in which nerves pierce muscle bellies along the route to their target tissues. Using ultrasound as a tool may be an effective method to identify whether patients present with piercing anatomy, which could be the cause of their symptoms.

### 4.5. Treatment Options for Occipital Neuralgia

Treatment of ON is a challenge but should be approached in a stepwise fashion in which conservative treatments are tried first and the more invasive techniques are considered only if conservative methods fail [3]. Conservative treatment for ON includes nerve blocks, electrical nerve stimulation, and medications, whereas more invasive approaches include surgical intervention [2].

In relation to conditions in which nerve entrapment and compression is a primary focus of potential symptoms, the use of botulism toxin has shown some promise [4,19,20]. The botulism toxin has been reported to be effective in the short-term relief of “potential” GON entrapment sites in symptomatic patients [4]. The botulism toxin could potentially provide prolonged relief in patients with ON when they are injected at a “specific” entrapment location rather than a “potential” site of entrapment [4]. This points to the potential use of botulism toxin to relieve symptoms in patients who present with symptoms resulting from the anatomic morphology reported in our study.

Just as nerve blocks have been employed to aid in the diagnosis of ON, the local anesthetic nerve block of the GON has been shown to be the most efficient therapeutic tool for treating ON [16,17]. As stated above, there is no standardized approach to performing this nerve block, which has led to a growing field of research investigating the most reliable and accurate methods for this procedure [17]. Many clinicians use a conventional approach that relies on superficial anatomic landmarks to locate the nerves and aid in identifying the location to administer the anesthetic, while some clinicians also use fluoroscopy to confirm the location of certain landmarks [18]. These approaches may be ambiguous and pose a risk of anesthetizing adjacent structures or injecting into adjacent vasculature such as the occipital artery [18]. Research into the risk associated with these injections is limited, but a complication rate of 5–10% has been reported, including headache, blurred vision, dizziness, and syncope [18]. Being able to better identify the location of specific nerves will not only reduce the number of complications due to this procedure but will also increase positive outcomes for patients who are treated correctly. 

Due to the ambiguity of using anatomical landmarks as an identifier for these nerves, there has been an increasing interest in using ultrasound to guide these nerve blocks. Ultrasound-guided nerve blocks have been shown to result in improved patient outcomes [18]. This result may be due to a more accurate injection of the nerves, which has been the subject of increased research interest, but also the use of a smaller quantity of anesthetic in these procedures [18]. An ultrasound-guided GON blockade at the level of the superior nuchal line has shown to be successful, resulting in improved pain scores compared to non-guided injections [18,21,22,23]. Injection at the C2 spinal nerve root, a more proximal location near the point of compression, may also result in an improved analgesic effect [18]. 

In a study focusing on the LON as the cause of the occipital pain, the best location of injection was concluded to be a region approximately 3 cm in diameter centered at a point 6.5 cm from midline and 5.3 cm inferior to the line between the external auditory canals [13]. This location is located more proximally than previously reported in prior studies and therefore thought to be a preferable location for injection [13]. A study investigating the role of the GAN in paroxysmal ear pain reported that blocking the GAN along with the roots of spinal nerves C2 and C3 resulted in improved relief of paroxysmal earaches as well as background pain compared to just blocking the GAN alone [15]. The overarching conclusions of these studies is that nerve blocks are an effective way to reduce pain in patients experiencing ON and other types of occipital pain, but that the efficacy of this treatment depends on correctly identifying and treating the root cause of the problem. Giving clinicians a better understanding of the numerous locations and types of nerve compression and entrapment and the concomitant symptoms can facilitate their ability to decide on a treatment, including the best injection locations for nerve blocks. 

Nerve blocks, although shown to be effective, are not the only method of treatment for patients with ON or other headache disorders. Occipital neurostimulation (ONS) or greater occipital peripheral nerve stimulation has been shown to potentially help manage a variety of headache disorders including ON, while offering a minimally invasive, relatively low risk, nerve preserving, reversible approach [4,5]. ONS was first introduced in 1999 as an option to treat refractory pain in patients, but larger studies have demonstrated its effectiveness in treating a wide variety of headache disorders, including but not limited to chronic and episodic migraine, cluster headache, occipital neuralgia, and trigeminal neuralgia [24]. ONS is the process in which fixed-frequency electric pulses are provided to distal branches of the GON and LON, providing potentially long-term benefits to patients with migraines [24]. A recent study reported that the majority of patients treated with ONS saw improvements in migraine severity, frequency, sleep quality, social or work activities, and the use of medication intake [24]. Although ONS has shown efficacy in pain reduction, it is a difficult procedure to perform [4]. The depth and level of lead placement is crucial to the success of the treatment, and dysesthesias in the overlying skin or painful muscle spasms may occur if the lead is placed incorrectly [4]. Ultimately, ONS has been supported as a treatment option for medically refractory ON, as reported by a recent systematic review of the literature [5]. 

If more conservative treatments such as botulism toxin injections, nerve blocks, or ONS fail to produce symptom relief in patients, then a more invasive approach such as surgery may be required. Neurolysis of the GON root may provide short-term pain relief in certain patients [1]. Factors such as tenderness over the GON, positive response to anesthetic blockade of the GON, history of direct occipital trauma, and pre-operative care under a neurologist or pain specialist have been suggested to correlate with positive outcomes of neurolysis [1]. However, the benefit of neurolysis in long-term pain relief has been questioned, as one study reported that up to 92% of patients experienced recurrence of symptoms within the first year [1,25].

Treatment for ON and the vast amount of other headache disorders has proven to be difficult but there are a variety of options for patients to choose from depending on their root cause, symptomatology, and anatomic presentation.

## 5. Conclusions

This study analyzed a unique presentation of the lesser occipital nerve (LON) and spinal accessory nerve (SAN) fusing as they pierce the sternocleidomastoid muscle (SCM). We also quantified the frequency of the LON and GAN piercing the SCM in our cadaveric sample. All of these anatomic presentations provide an example of potential nerve compression and therefore potential for neuralgic symptoms in patients. We hypothesize that some cases of occipital neuralgia may result from an undiagnosed case of LON/SAN compression by the SCM. Occipital neuralgia and many other headache disorders are difficult to accurately diagnose due to the vast plexuses of nerves in the region, the multiple locations of potential nerve compression, and the variety of other contributing factors that if treated alone may not completely resolve the problem. It is important to continue to identify potential locations of compression and reasons for nerve pain in order to aid clinicians during the process of finding the correct diagnosis and treatment. We hope our study shines light on additional potential anatomic explanations for neuralgic symptoms in patients, which may help clinicians to identify these presentations and treat them accordingly.

## Figures and Tables

**Table 1:** Data on the number of anatomical specimens identified with each nerve variant, broken down by sex (M = males, F = females). Abbreviations: GAN = greater auricular nerve; LON = lesser occipital nerve; SAN = spinal accessory nerve; SCM = sternocleidomastoid.

Number of Presentations	SAN Fusing w/LON, Piercing SCM	LON Piercing SCM Alone	GAN Piercing SCM Alone	LON and GAN Piercing SCM
Number of Presentations	4 (4F)	68 (27F/41M)	29 (12F/17M)	25 (11F/14M)
Percentage (%)	3.77%	58.1%	25.9%	24.5%
Female	55	49	51	42
Male	51	68	61	60
Total	106	117	112	102

## Data Availability

All relevant data are provided within the paper.
